# Toosendanin Induces Hepatotoxicity by Facilitating ALOX5-Mediated Lipid Peroxidation and Sensitizing Cells to Ferroptosis

**DOI:** 10.3390/ph18071078

**Published:** 2025-07-21

**Authors:** Jiajie Ni, Liru Huang, Yifan Tian, Changxin Zhao, Ziyi Zhou, Feihai Shen, Zhiying Huang

**Affiliations:** 1 School of Pharmaceutical Sciences, Sun Yat-sen University, Guangzhou 510006, China; nijj6@mail2.sysu.edu.cn (J.N.); huanglr25@mail2.sysu.edu.cn (L.H.); tianyf3@mail2.sysu.edu.cn (Y.T.); zhaochx33@mail2.sysu.edu.cn (C.Z.); zhouzy87@mail2.sysu.edu.cn (Z.Z.); 2 School of Pharmacy, Guangdong Pharmaceutical University, Guangzhou 510006, China

**Keywords:** toosendanin, hepatotoxicity, 5-lipoxygenase, lipid peroxidation, ferroptosis

## Abstract

**Background**: *Fructus Meliae Toosendan* (FMT) is a traditional Chinese medicine used to treat ascariasis; however, its reported hepatotoxicity limits its application. Toosendanin (TSN), as a principal active component, is recognized as the primary toxic ingredient responsible for FMT-induced hepatotoxicity, but the underlying mechanisms remain elusive. **Methods**: HepG2 cells were treated with TSN and analyzed using Western blotting and qPCR assays for related gene transcription and protein expression. Lipid peroxidation and ferroptosis markers were measured. Balb/c and C57BL/6 mice received various doses of TSN administration, and their liver function was assessed with serum biochemistry and histopathology. Network pharmacology and oxidative lipidomics were performed to identify key targets and metabolites. **Results:** TSN triggered ferroptosis both in vitro and in vivo, accompanied by the elevated expression of 5-lipoxygenase (ALOX5) and its downstream metabolites. The ALOX5 level modulated hepatocyte sensitivity to TSN-induced ferroptotic damage. An ALOX5 knockdown alleviated TSN-induced liver injury and ferroptosis in vivo. **Conclusions**: Our study demonstrated that TSN induces hepatotoxicity by facilitating ALOX5-mediated lipid peroxidation, thereby sensitizing cells to ferroptosis.

## 1. Introduction

Herb-induced liver injury (HILI) has become a public health issue, contributing to 26.81% of the drug-induced liver injury (DILI) cases linked to traditional Chinese medicine (TCM) or herbal and dietary supplement (HDS) products [1,2]. Elucidating the mechanistic basis of HILI is therefore critical for ensuring the safe clinical application of herbal medicines. *Fructus Meliae Toosendan* (FMT), the ripe fruit of *Melia toosendan* Sieb. et Zucc., is used to regulate *qi*, soothe the liver in TCM theory, and expel digestive tract parasites. FMT was first recorded in Shen Nong’s Herbal Classic and is used as a mildly toxic Chinese medicine [3,4]. Toosendanin (TSN) has been identified as the main toxic ingredient in FMT [3,5], and is designated as a quality control marker according to the *Chinese Pharmacopoeia* (2020 edition). TSN possesses pharmacological properties such as anti-botulinum [6], antiviral [7], and antitumor [8] properties, suggesting its therapeutic potential as a herbal medicine. However, TSN-induced liver injury limits its clinical application. Several studies have explored the potential mechanisms underlying TSN-induced hepatotoxicity, including oxidative stress [9], autophagic disruption [10], and impaired lipid metabolism. Our previous evidence demonstrated that TSN could induce lipid metabolism disorder by inhibiting the liver X receptor α (LXRα)/lipin 1/sterol regulatory element-binding protein 1 (SREBP1) signaling pathway, contributing to HILI [11]. However, the precise molecular mechanisms responsible for TSN-mediated hepatotoxicity have not yet been fully elucidated.

Ferroptosis, an iron-dependent form of cell death characterized by lipid peroxide accumulation, is regulated through lipid metabolism pathways. It is primarily accompanied by the depletion of glutathione (GSH) and glutathione peroxidase 4 (GPX4) [12]. Ferroptotic hepatocellular damage driven by iron-dependent lipid peroxidation plays a pivotal role in the pathogenesis of DILI [13]. Intracellular iron accumulation and lipid peroxidation are two central biochemical events leading to ferroptosis [14]. As a core process of ferroptosis, lipid peroxidation involves irreversible oxidative damage to polyunsaturated fatty acid phospholipids, disrupting the cell membrane and causing cell death [15]. Our previous research showed that TSN-induced hepatotoxicity involves intracellular iron overload and ferroptotic cell death [16]; however, the specific signaling pathway mediating iron-dependent lipid peroxide accumulation remains uncharacterized. Thus, further investigation is needed to elucidate how TSN induces ferroptosis through lipid peroxidation.

Ferroptosis-associated lipid peroxidation results from both iron-catalyzed autoxidation and lipoxygenase (ALOX)-mediated enzymatic oxidation [15]. ALOXs are large non-heme iron-containing enzymes, and the Fe^2+^ requires oxidation to Fe^3+^ for activation, which directly promotes the synthesis of lipid radicals [14,17]. ALOXs predominantly catalyze the oxidation of polyunsaturated fatty acids (PUFAs), directly targeting arachidonic and adrenic acid residues in phosphatidylethanolamine to induce ferroptosis [18]. Mammalian ALOXs have six isoforms, which catalyze different lipid compounds. 5-Lipoxygenase (ALOX5) catalyzes the conversion of arachidonic acid (AA) to its unstable metabolic intermediate, 5-hydroperoxyeicosatetraenoic acid (5-HPETE) [19], which is metabolized to 5-hydroxyeicosatetraenoic acid (5-HEPE), a lipid peroxidation inducer. The inhibition of ALOX5 has been reported to attenuate the accumulation of lipid peroxides and neuronal damage [20], as well as to protect mice against acetaminophen-induced liver injury [21]. However, whether ALOX5-mediated lipid peroxidation contributes to TSN-induced hepatotoxicity remains unknown.

This study investigated TSN-induced liver injury by investigating its mechanisms in triggering lipid peroxidation and ferroptosis.

## 2. Results

### 2.1. TSN Induces Hepatotoxicity via Ferroptosis In Vitro

**Figure 1:**
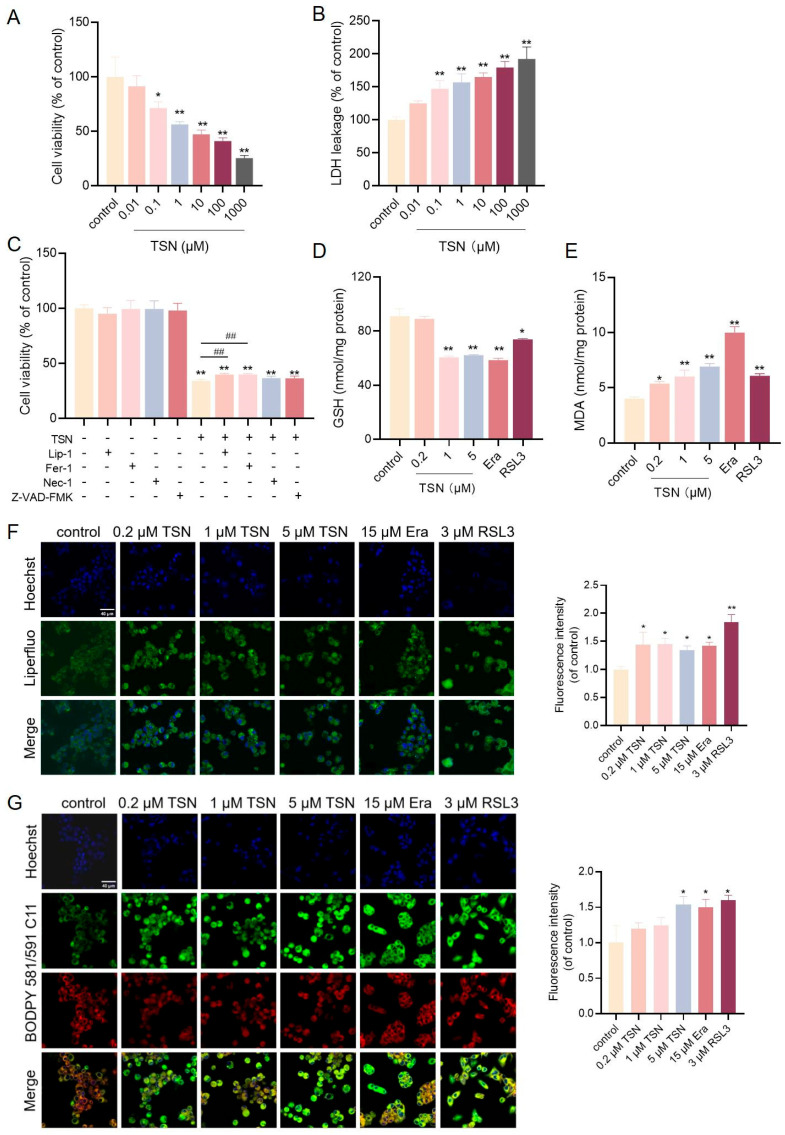

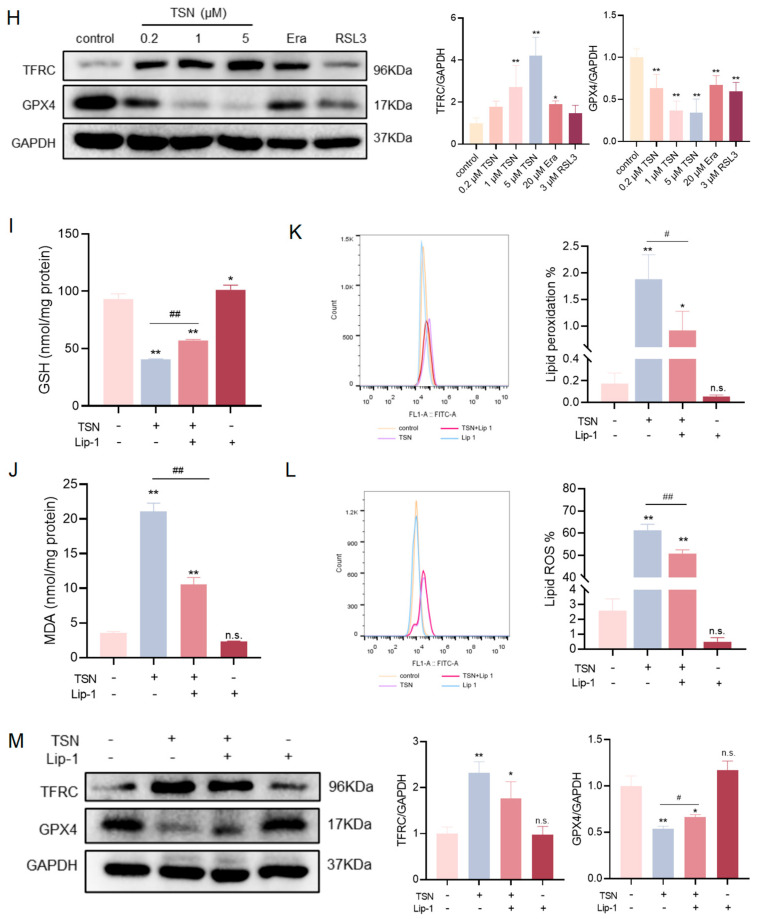
TSN induces hepatotoxicity via ferroptosis in vitro. (**A**,**B**) HepG2 cells were treated with TSN at various concentrations (0, 0.01, 0.1, 1, 10, 100, or 1000 μM) for 48 h. (**A**) Cell viability was measured by a CCK-8 assay. (**B**) LDH leakage was measured by an LDH assay kit. (**C**) The CCK-8 assay of cell viability in HepG2 cells after treatment with TSN (5 μM) ± indicated inhibitors (2 μM liproxstatin-1, 4 μM ferrostatin-1, 2 μM necrostatin-1, or 5 μM Z-VAD-FMK) for 48 h. (**D**,**E**) HepG2 cells were treated with TSN at various concentrations (0.2, 1, or 5 μM) and ferroptosis inducers (10 μM erastin and 2.5 μM RSL3) for 48 h. The levels of GSH and MDA were measured by GSH and MDA assay kits. (**F**,**G**) After using the Liperfluo probe and BODIPY 581/591 C11, the cells were inspected using confocal microscopy, and the fluorescence intensity was analyzed by the Image J software (version 1.53t). Scale bar: 40 μm. (**H**) HepG2 cells treated with TSN (0.2, 1, or 5 μM), erastin (15 μM), and RSL3 (3 μM) for 48 h were analyzed by Western blotting for the protein levels of TFRC and GPX4, which were standardized based on the respective level of GAPDH. (**I**–**M**) HepG2 cells were treated with TSN (5 μM) ± Lip-1 (2 μM) for 48 h. (**I**,**J**) The levels of GSH and MDA were measured by GSH and MDA assay kits, respectively. (**K**,**L**) The accumulation of lipid peroxide and lipid ROS were measured using the Liperfluo probe and BODIPY 581/591 C11, and determined by flow cytometry. (**M**) The protein levels of TFRC and GPX4 were analyzed by Western blotting. n.s. (not significant); * *p* < 0.05 and ** *p* < 0.01 versus vehicle control; # *p* < 0.05 and ## *p* < 0.01 versus TSN group.

HepG2 cells were treated with TSN for 48 h to assess TSN’s cellular toxicity. As shown in Figure 1A, TSN reduced the cell viability in a concentration-dependent manner with an estimated IC_50_ value of 8.3 µM. Figure 1B shows that TSN exposure elevated LDH leakage. HepG2 cells were treated with TSN only or TSN combined with different cell death inhibitors, such as the ferroptosis inhibitors Lip-1 and Fer-1, the necroptosis inhibitor Nec-1, and the apoptosis inhibitor Z-VAD-FMK. The CCK-8 results showed that only Lip-1 and Fer-1 prevented TSN-induced cell death (Figure 1C). The ferroptosis inducers RSL3 and Era and the ferroptosis inhibitor Lip-1 were used as experimental interventions. GSH serves as an important regulator of intracellular redox homeostasis. The GSH level was significantly reduced after incubation with TSN, RSL3, or Era (Figure 1D), whereas the ferroptosis inhibitor Lip-1 reversed the TSN-induced reduction in GSH (Figure 1I). Lipid peroxidation was evaluated with the fluorescent probes BODIPY 581/591 C11 and Liperfluo, and by measuring the malondialdehyde (MDA) concentrations. These studies showed that the TSN, RSL3, and Era groups significantly induced lipid peroxidation (Figure 1E–G), which was effectively mitigated by co-treatment with Lip-1 (Figure 1J–L). Ferroptosis-related proteins were determined by Western blotting after treatment with TSN or the ferroptosis inducers RSL3 and erastin (Era). Decreased GPX4 and increased TFRC levels were observed, suggesting TSN-induced ferroptosis activation (Figure 1H) and a protective effect of ferroptosis inhibitor Lip-1 against TSN (Figure 1M). Together, these data support the idea that TSN promoted ferroptosis with lipid peroxidation, thereby inducing hepatotoxicity.

### 2.2. TSN Induces Ferroptosis In Vivo

**Figure 2:**
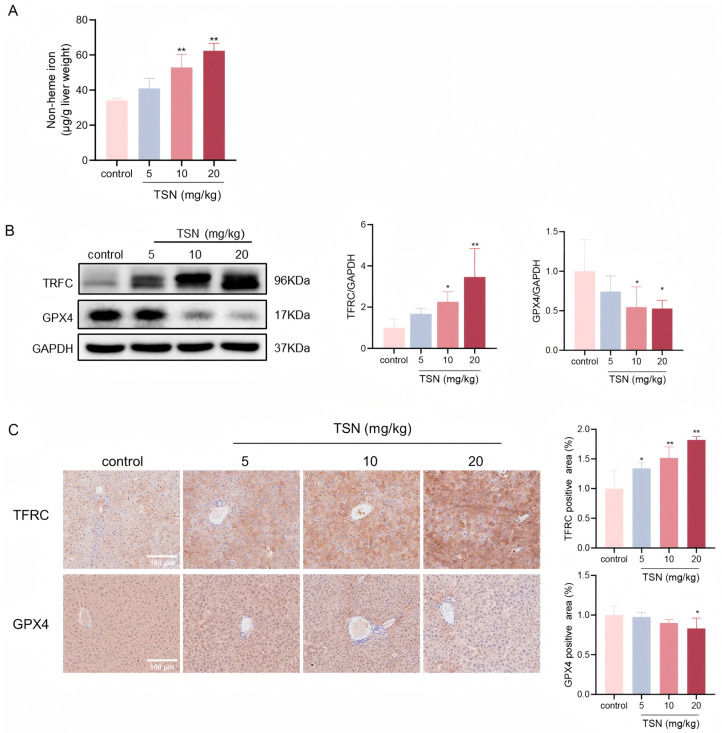
TSN induced ferroptosis in vivo. Balb/c mice were intraperitoneally administered TSN at different doses (5, 10, or 20 mg/kg/d) or a vehicle for 7 days (*n* = 6). (**A**) Non-heme iron was measured in the liver tissues. (**B**) The protein levels of TFRC and GPX4 were analyzed by Western blotting. (**C**) Representative immunohistochemistry (IHC) images of TFRC and GPX4. Scale bar: 150 μm. * *p* < 0.05 and ** *p* < 0.01 versus vehicle control.

To further investigate the effect of TSN-induced ferroptosis on the liver, Balb/c mice were used in vivo. In our previous research, a TSN treatment increased the serum levels of ALT, AST, and LDH and caused hepatic damage [11]. As shown in Figure 2A, the 10 and 20 mg/kg TSN treatments caused an increase in the non-heme iron content. Western blotting revealed decreased GPX4 and increased TFRC levels after TSN administration (Figure 2B). The analysis demonstrated strong GPX4 staining and weak TFRC immunoreactivity in control hepatocytes, whereas TSN-treated mice exhibited marked dose-dependent GPX4 depletion and concomitant TFRC upregulation, consistent with the Western blotting results (Figure 2C).

### 2.3. TSN Promotes the Expression of ALOX5 and Its Downstream Metabolites In Vitro

**Figure 3:**
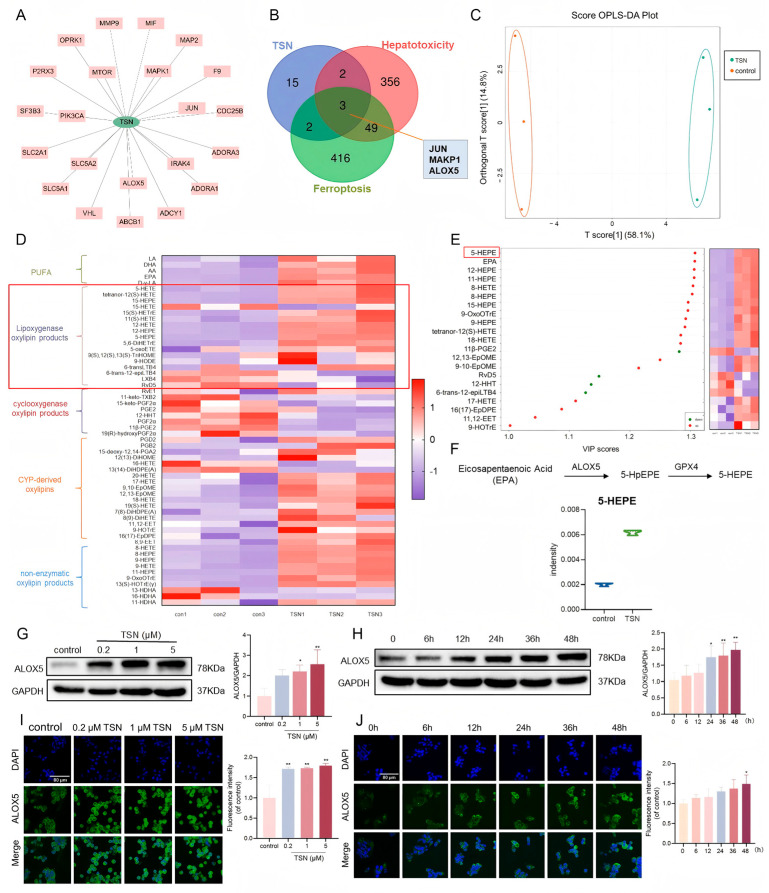
TSN promotes the expression of ALOX5 and its downstream metabolites in vitro. (**A**) The compound–target network for TSN. The pink node represents the targets. (**B**) A Venn graph of genes in TSN, ferroptosis, and hepatotoxicity. (**C**–**F**) HepG2 cells were treated with TSN (5 μM) for 48 h (*n* = 3). (**C**) Orthogonal partial least-squares discriminant analysis (OPLS–DA) score plot. There was a clear distinction between the control and TSN–treated groups. (**D**) Heatmap showing the expression of metabolites involved in the polyunsaturated fatty acid (PUFA) metabolism pathway. The red box shows the levels of LOX metabolites. (**E**) Important volatiles (VIP > 1.0) identified by OPLS–DA. The heatmap (right) indicates the relative concentration of the corresponding metabolite in the respective groups. The red box shows the metabolite with the highest VIP values. (**F**) The content changes in 5–HEPE between different groups. (**G**,**H**) The protein level of ALOX5 was analyzed by Western blotting. (**I**,**J**) Immunofluorescence images of the ALOX5 protein level. Scale bar: 80 μm. The data are presented as the mean ± SD of three independent experiments. * *p* < 0.05 and ** *p* < 0.01 versus vehicle control.

The “component–target” network of TSN (Figure 3A) was constructed by the software of Cytoscape 3.7.2, presenting TSN and its 22 target proteins. As shown in Figure 3B, the Venn diagram identified three overlapping targets (JUN, MAPK1, and ALOX5) linking TSN exposure, hepatotoxicity, and ferroptosis pathogenesis. Given that ALOXs are known to be involved in the pathway modulation of ferroptosis, we investigated the lipid metabolites in HepG2 cells after TSN treatment using oxidative lipidomics techniques. The partial least-squares discriminant analysis indicated significant metabolite alterations following a 48 h TSN treatment (Figure 3C). The heatmap demonstrated progressive elevation in the ALOX metabolite levels, including 5-hydroxyeicosapentaenoic acid (5-HEPE), 12-hydroxyeicosatetraenoic acid (12-HETE), and 15-hydroxyeicosapentaenoic acid (15-HEPE) (Figure 3D). The VIP (variable importance in projection) value is an analytical expression of the weighted sum of squared variations across each dimension in the orthogonal partial least-squares discriminant analysis (OPLS-DA). In Figure 3E, the volatile compounds with VIP values > 1.0 are shown. 5-HEPE was identified as the most significant metabolite based on the VIP analysis. It was mainly derived from the 5-lipoxygenase enzyme. The specific change in 5-HEPE is shown in Figure 3F, showing an increase in the 5-HEPE content after the TSN treatment. The Western blot showed that the ALOX5 expression level was increased after the TSN (0.2, 1, or 5 μM) treatment for 48 h (Figure 3G) or the 5 μM TSN treatment for different times (0, 6, 12, 24, 36, or 48 h) (Figure 3H). Those trends were also confirmed by immunofluorescence (Figure 3I,J). Collectively, these results indicate that TSN triggers ALOX5-driven lipid peroxidation, promoting ferroptosis susceptibility.

### 2.4. The Level of ALOX5 Affects the Sensitivity of TSN-Induced Cell Damage Involving Ferroptosis

**Figure 4:**
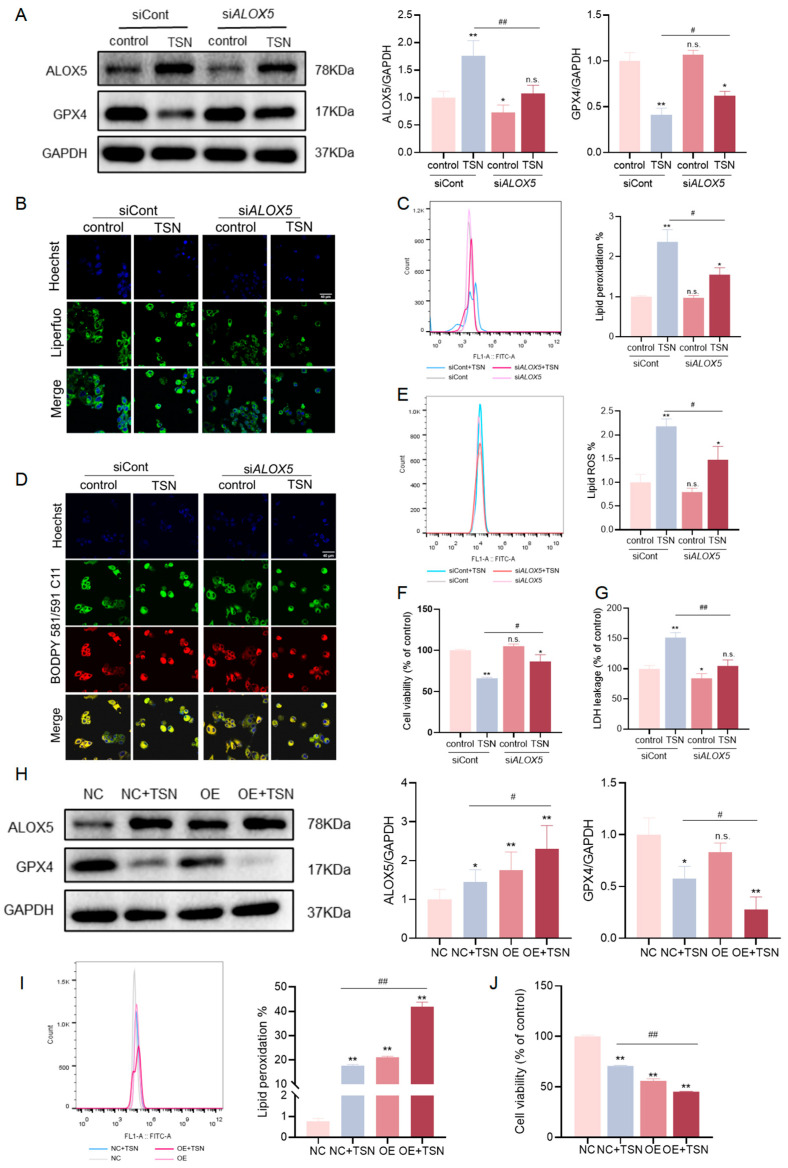
The level of ALOX5 affected the sensitivity of TSN-induced cell damage involving ferroptosis. (**A**–**G**) HepG2 cells were transfected with control or *ALOX5* siRNA and cultured for 48 h before treatment with TSN (5 μM) for 48 h. (**A**) The protein levels of ALOX5 and GPX4 were analyzed by Western blotting. (**B**,**C**) The accumulation of lipid peroxide in HepG2 cells was detected with the Liperfluo probe, and then analyzed by confocal imaging and flow cytometry. Scale bar: 40 μm. (**D**,**E**) The accumulation of lipid ROS in HepG2 cells was detected with BODIPY 581/591 C11, and then analyzed by confocal imaging and flow cytometry. Scale bar: 40 μm. (**F**) The cell viability was determined by a CCK-8 assay. (**G**) LDH leakage was measured by an LDH assay kit. (**H**–**J**) HepG2 cells were transfected with a vector or ALOX5 plasmid for 48 h and then treated with TSN (5 μM) for 48 h. (**H**) The protein levels of ALOX5 and GPX4 were analyzed by Western blotting. (**I**) The accumulation of lipid peroxide in HepG2 cells was detected with the Liperfluo probe and analyzed by flow cytometry. (**J**) The cell viability was determined by a CCK-8 assay. The data are presented as the mean ± SD of three independent experiments. n.s. (not significant); * *p* < 0.05 and ** *p* < 0.01 versus vehicle control; # *p* < 0.05 and ## *p* < 0.01 versus TSN group.

To determine the role of ALOX5 in TSN-induced ferroptosis, an *ALOX5*-specific siRNA knockdown was performed. As expected, the protein level was decreased by the corresponding siRNA (Figure 4A). In the si*ALOX5* group, a Western blot analysis revealed a higher GPX4 protein level compared to the TSN group (Figure 4A). In addition, the knockdown of ALOX5 significantly mitigated lipid peroxide (Figure 4B,C) and lipid ROS (Figure 4D,E) accumulation upon treatment with TSN. The cell viability and the rate of LDH leakage were partially restored (Figure 4F,G) in TSN-treated HepG2 cells. Taken together, the downregulation of ALOX5 reduced lipid peroxidation, thereby attenuating TSN-induced ferroptosis and increasing the cell survival rate.

To further explore whether the exogenous overexpression of ALOX5 enhanced TSN-induced lipid peroxidation and promoted ferroptosis, we transfected overexpression plasmids into HepG2 cells. The Western blot results confirmed that the ALOX5 level was successfully elevated (Figure 4H). The overexpression of ALOX5 exacerbated the TSN-induced decrease in the GPX4 protein level (Figure 4H) and enhanced the lipid peroxidation induced by TSN (Figure 4I). After the TSN treatment, cell death was enhanced in HepG2 cells in the ALOX5-overexpression group compared to the NC group (Figure 4J).

These data suggest that ALOX5 is a crucial protein in lipid peroxidation-dependent ferroptosis caused by TSN in HepG2 cells.

### 2.5. ALOX5 Downregulation Attenuates TSN-Induced Hepatic Injury In Vivo

**Figure 5:**
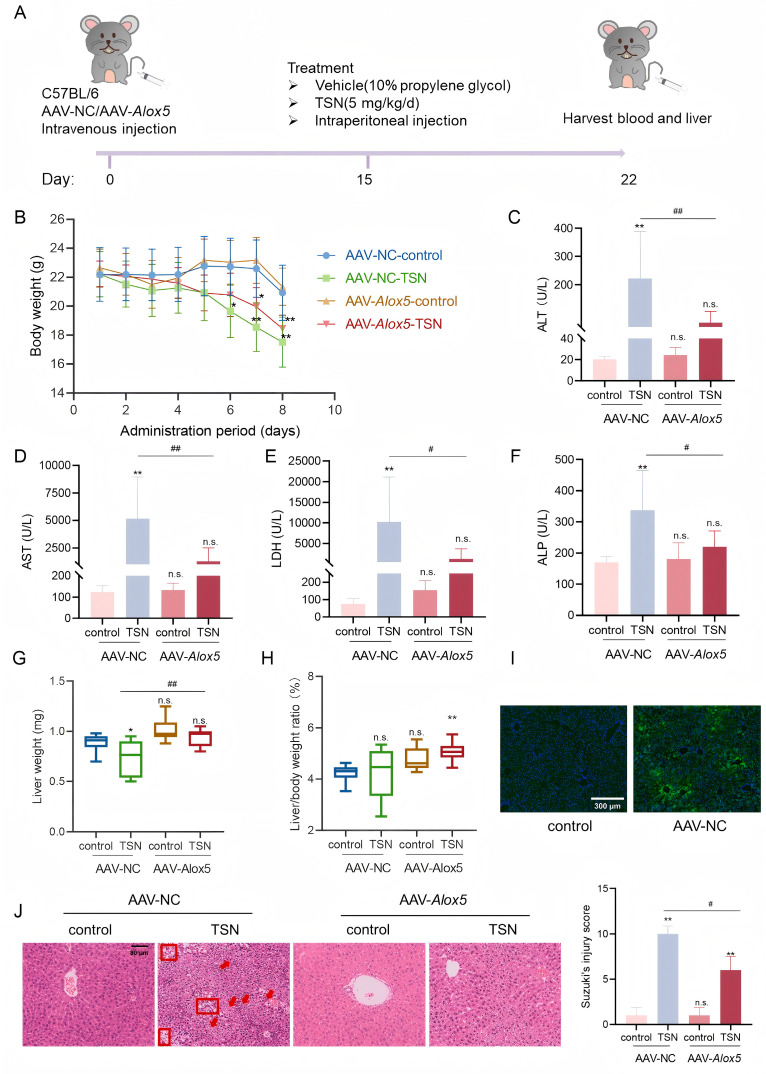
ALOX5 downregulation attenuated TSN-induced hepatic injury in vivo. (**A**) Schematics illustrating C57BL/6 mouse treatment. C57BL/6 mice were injected with AAV8 downregulating ALOX5 via the tail vein, and were then intraperitoneally administered TSN (5 mg/kg) or a vehicle for 7 days. The individual body weight was recorded every day until day 8. Serum and liver tissue were harvested after the TSN treatment. (**B**) The body weight of the mice exposed to TSN (5 mg/kg) for 7 days. The effect of TSN on the levels of ALT (**C**), AST (**D**), LDH (**E**), and ALP (**F**) in serum was detected. (**G**) The liver weight of the mice. (**H**) The ratio of the liver weight to the body weight. (**I**) An immunohistological analysis of the AAV transduction efficiency. AAV-transduced livers expressed EGFP (green). Scale bar: 300 μm. (**J**) The histological changes in mouse livers were indicated by H&E staining. Scale bar: 80 μm. Liver injury was scored using the Suzuki criteria. The data are presented as the mean ± SD. n.s. (not significant); * *p* < 0.05 and ** *p* < 0.01 versus vehicle; # *p* < 0.05 and ## *p* < 0.01 versus AAV-NC-TSN group; *n* = 10.

To investigate the role of ALOX5 in TSN-induced hepatic injury in vivo, C57BL/6 mice received an intravenous adeno-associated viral serotype 8 (AAV8)-mediated ALOX5 knockdown (Figure 5A). Following a 2-week stabilization period post-model establishment, the mice were intraperitoneally administered 5 mg/kg TSN or a vehicle for 7 days. After the TSN treatment, the mice exhibited fur discoloration, a decreased vitality, and body weight loss, with an attenuated weight reduction in the AAV-*Alox5*-TSN group versus the AAV-NC-TSN controls (Figure 5B). Liver function tests on the mice demonstrated that ALT, AST, LDH, and ALP were elevated in the TSN-treated mice and were significantly attenuated in the AAV-*Alox5*-TSN group (Figure 5C–F). Compared to the obvious liver weight decrease in AAV-NC-TSN mice, the AAV-*Alox5*-TSN group maintained a significantly higher hepatic mass (Figure 5G). However, while the liver-to-body-weight ratio showed no significant differences between the AAV-NC-TSN and AAV-*Alox5*-control groups compared to the AAV-NC-controls, the AAV-*Alox5*-TSN group exhibited a significantly elevated ratio (Figure 5H). An immunohistological analysis showed that large areas of AAV-transduced liver tissues were EGFP-positive (Figure 5I), indicating effective transduction. A histopathological analysis demonstrated that, in both control groups, the structure of the hepatic lobules was normal, the hepatocytes were orderly arranged around the central vein, and the hepatic sinusoids were well defined. In both TSN groups, a mussily arranged hepatic plate, hepatocellular swelling, and vacuolization were observed. Moreover, the AAV-NC-TSN group showed multiple areas of spotty necrosis (red arrows) and ballooning degeneration (red boxes). The AAV-*Alox5*-TSN group showed a clear hepatic sinusoid and partial swelling hepatocytes (Figure 5J). In comparison, the AAV-*Alox5*-TSN group had milder pathological damage.

### 2.6. ALOX5 Knockdown Protects Against TSN-Induced Ferroptosis In Vivo

**Figure 6:**
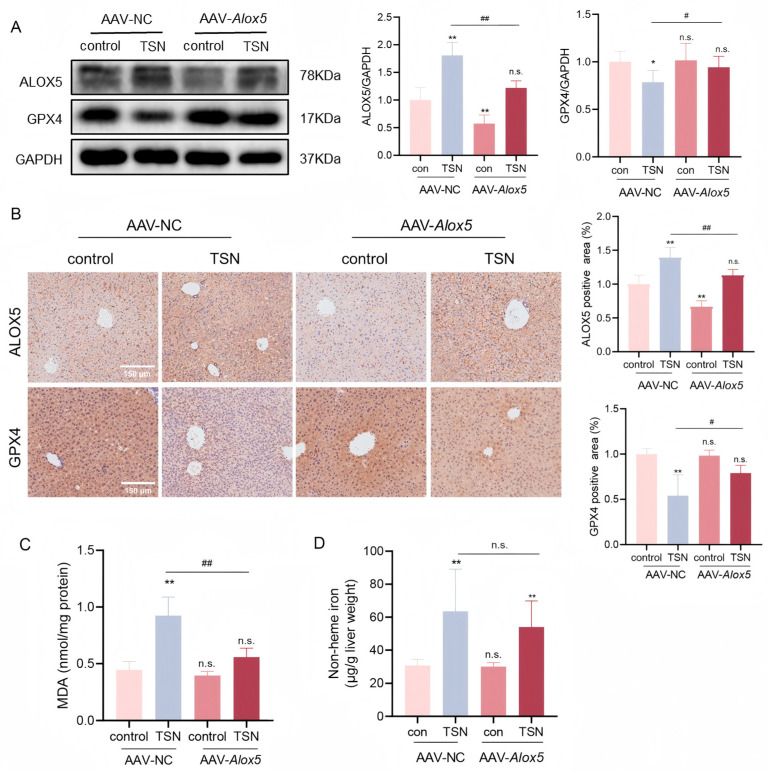
An ALOX5 knockdown protects against TSN-induced ferroptosis in vivo. (**A**) The expression levels of ALOX5 and GPX4 in liver tissues were analyzed by Western blotting. (**B**) Representative results of IHC staining for ALOX5 and GPX4. The percentage of positive area was analyzed using the Image J software (version 1.53t). Scale bar: 150 μm. (**C**) The level of MDA in liver tissues was detected using an MDA assay kit. (**D**) Non-heme iron was measured in liver tissues. The data are presented as the mean ± SD. n.s. (not significant); * *p* < 0.05 and ** *p* < 0.01 versus vehicle control; # *p* < 0.05 and ## *p* < 0.01 versus AAV-NC-TSN group; *n* = 6.

The ALOX5 knockdown efficiency was verified by a Western blot (Figure 6A) and IHC (Figure 6B). Notably, TSN-mediated GPX4 downregulation was partially reversed in AAV-*Alox5*-TSN mice relative to the AAV-NC-TSN group (Figure 6A). Similar results were presented by IHC (Figure 6B). The ALOX5 knockdown mitigated the TSN-induced increase in MDA production (Figure 6C). The mice administered TSN (5 mg/kg) had a higher non-heme iron content compared to the vehicle group (10% propylene glycol), and there was no significant difference between AAV-*Alox5* and AAV-NC mice (Figure 6D). The above data indicate that the ALOX5 knockdown reduced lipid peroxidation, thus alleviating TSN-induced ferroptosis in mouse livers.

## 3. Discussion

TSN is the primary toxic and active ingredient derived from *Melia toosendan* Sieb. et Zucc., a traditional Chinese herb used for over a millennium [3]. Recently, TSN has been used as an ascaricide and anti-botulism agent, but its hepatotoxicity has also been reported. However, the precise mechanisms of TSN-induced hepatotoxicity require further elucidation. In this study, we demonstrated the hepatotoxic effect of TSN involving ferroptosis with lipid peroxidation. The toxic targets of TSN were predicted by network toxicology, and we found that 5-HEPE (produced by ALOX5) accumulated in HepG2 cells treated with TSN, as detected by oxidative lipidomics. To explore the importance of ALOX5-mediated lipid peroxidation in TSN-induced ferroptosis, HepG2 cells and ALOX5-downregulated C57BL/6 mice were used in vitro and in vivo. The results showed that the downregulation of ALOX5 restored the GPX4 protein level and reduced lipid peroxide accumulation, thus attenuating TSN-caused ferroptosis and minimizing hepatocyte damage. Conversely, the overexpression of ALOX5 significantly exacerbated these effects, making HepG2 cells more susceptible to ferroptosis. Taken together, we discovered an unrecognized role of ALOX5 in TSN-caused hepatotoxicity.

A previous study described the phenomenon of TSN-induced intracellular iron overload [16] at 24 h, but did not elucidate the signaling pathway responsible for the iron-dependent accumulation of lipid peroxidation. We demonstrated that exposure to TSN induces pathological and biochemical alterations in liver tissues [11]. Although the HepG2 cell line is derived from tumor cells, it shares characteristics with normal human hepatocytes [22]. Therefore, the HepG2 cell line was utilized as an in vitro cellular model. In order to further determine the TSN-induced ferroptosis, the ferroptosis inducers RSL3 and erastin were used, and more lipid-peroxidation-related indicators were detected. The changes in the GSH level, lipid peroxide level, and ferroptosis-related protein levels after treatment with TSN or RSL3 and erastin were consistent, whereas Lip-1 reversed all these changes. Lip-1 is an inhibitor of ferroptosis that reduces the accumulation of lipid peroxides and ROS, rather than directly reducing the intracellular iron content [23]. The above results indicate that TSN induces lipid peroxidation and causes ferroptosis, leading to hepatotoxicity.

The peroxidation of lipids involves autoxidation and an enzyme-mediated process. It should be noted that some enzymes, such as ALOXs, not only catalyze free PUFAs, but also catalyze membrane phospholipids and cause ferroptosis [15]. Therefore, we analyzed the PUFA metabolites to investigate the mechanism of lipid peroxidation through oxidative lipidomics. The incorporation of ALOX substrates has been demonstrated to expedite the process of cell death [24]. OPLS-DA was performed to maximize inter-group differentiation and obtain the significant metabolites. The VIP value is an analytical expression of the weighted sum of the squared variations across each dimension in the OPLS-DA analysis. 5-HEPE had the highest VIP value, and thus, was identified as the most significant metabolite. ALOX5 mediates the conversion of EPA to 5-HEPE [25]. ALOX5 was considered to be the critical enzyme in TSN-induced lipid peroxidation and caused ferroptosis according to the results of oxidative lipidomics, which was consistent with the results of the network toxicology study.

In addition, the increase in downstream metabolites indicated elevated ALOX5 enzyme activity. ALOXs directly catalyze the generation of lipid radicals through non-heme iron in their catalytic region [14]. TFRC expression and the content of non-heme iron were increased after TSN administration in Balb/c mice, suggesting that TSN promotes iron uptake, increases intracellular iron, and thereby stimulates 5-lipoxygenase activity.

TSN increased the protein level of ALOX5 in a concentration- and time-dependent manner. To determine the role of ALOX5 in TSN-induced lipid peroxidation and ferroptosis, HepG2 cells and C57BL/6 mice were employed as in vitro and in vivo models, respectively. It has been reported that Balb/c mice clear AAV-transduced cells more rapidly than C57BL/6 mice, while C57BL/6 mice exhibit a longer persistence of therapeutic protein expression after AAV transduction into liver cells [26]. Since this study aimed to investigate the role of ALOX5 in TSN-induced hepatotoxicity in vivo, C57BL/6 mice were selected to achieve a higher knockdown efficiency. The dose of 5 mg/kg of TSN was chosen to establish the TSN-induced liver injury model based on the results of the acute toxicity studies in Balb/c mice. The results indicated that the knockdown of ALOX5 restored the GPX4 protein level, reduced lipid peroxide accumulation, and maintained a balance between ferroptosis monitoring and lipid peroxidation after the TSN treatment. In general, the downregulation of ALOX5 alleviated TSN-induced ferroptosis and reduced liver damage. The overexpression of ALOX5 markedly sensitized HepG2 cells to ferroptosis and aggravated hepatotoxicity. The serum iron level and non-heme iron content in the liver tissues showed no significant difference between AAV-*Alox5* and AAV-NC mice, whereas the MDA levels were more prominent in AAV-NC mice than in ALOX5 knockdown mice. This suggests that, in TSN-induced ferroptosis, the ALOX5 level contributes to lipid peroxidation more significantly than an iron overload. In general, this study demonstrates the pivotal role of ALOX5-driven lipid peroxidation in mediating TSN-induced ferroptosis and hepatotoxicity.

## 4. Materials and Methods

### 4.1. Reagents and Chemicals

TSN (purity > 98%, MB6571) was acquired from Meilunbio (Dalian, China), and the structure was elucidated (Supplementary Figures S1 and S2). Erastin, RSL3, liproxstatin-1, and ferrostatin-1 were purchased from MedChemExpress (Monmouth Junction, NJ, USA). Necrostatin-1 and Z-VAD-FMK were purchased from Selleck Chemicals (Houston, TX, USA).

### 4.2. HepG2 Cell Culture and Transfection

HepG2 cells were acquired from the cell bank of the Chinese Academy of Sciences (Shanghai, China), and were cultured in high-glucose DMEM supplemented with 10% FBS. HepG2 cells at a 70% density were transfected using Lipofectamine 3000 (L3000015, Invitrogen, Carlsbad, CA, USA) according to the reagent instructions. ALOX5-targeting siRNA and scrambled control siRNA were dissolved in solution at a concentration of 20 μM. The pT7-6xHis-*ALOX5* (P52010) plasmid and the pET-28a vector (P0023) were purchased from MiaoLingBio (Wuhan, China). Following 6 h of transfection, the culture medium was changed and the cells were cultured for an additional 42 h before the analysis. The sequences of siRNAs are enumerated in Supplementary Table S1.

### 4.3. Cell Viability and Cytotoxicity Assay

The Cell Counting Kit-8 assay (Bimake, Houston, TX, USA) and the LDH assay kit (HY-K1090, MedChemExpress, USA) were utilized to evaluate cell viability. HepG2 cells were plated in 96-well plates at a density of 6 × 10^3^ per well and analyzed in accordance with the provided protocol. In brief, 10 μL of the CCK-8 reagent was added to each well after treatment, as described in the figure legends. The plates were incubated for another 1.5 h at 37 °C and the optical densities were read at a 450 nm wavelength. For the cytotoxicity assay, 50 μL of cell culture supernatant was collected from each well, mixed with 50 μL of working solution, and incubated for 30 min at room temperature. The absorbance at 490 nm was recorded after the addition of a stop solution. The absorbance readings were taken using a microplate reader (Thermo Multiskan Sky, Waltham, CA, USA).

### 4.4. Western Blotting

Liver tissues or HepG2 cells lysates were homogenized using cell lysis buffer (Beyotime, Shanghai, China), which included 1% phenylmethanesulfonyl fluoride (PMSF) and 2% phosphatase inhibitors. For each group, 30 μg of protein samples were prepared and processed through SDS-PAGE, after which they were transferred to a PVDF membrane (Merck Millipore, Billerica, MA, USA) and then blocked with skim milk at room temperature for 1 h. After overnight incubation at 4 °C with the primary antibodies (1:1000), the membranes were treated with the appropriate secondary antibodies (1:5000) for 1 h at room temperature. The antibody details are listed in Supplementary Table S2. The developer solution preparation followed the Enhanced Chemiluminescent kit (NCM Biotech, Suzhou, China) instructions. The protein bands were visualized using a chemiluminescence detection system (BioRad Laboratories, Hercules, CA, USA). ImageJ was used to analyze the gray values.

### 4.5. Quantitative Real-Time PCR (RT-qPCR)

The total RNA was extracted by following the guidelines provided by the RNASimple Total RNA Kit (Tiangen Biotech, Beijing, China). The RNA concentration and purity were determined using a NanoDrop 2000 spectrophotometer (Thermo Fisher Scientific, Waltham, CA, USA). RNA reverse transcription was performed with an M-MLV reverse transcription kit (Accurate Biology, Hunan, China). For real-time PCR, the SYBR Green Premix Pro Taq HS qPCR Kit (Accurate Biology, Hunan, China) was used. The primer sequences (Sangon Biotech, Shanghai, China) are listed in Supplementary Table S3. The relative mRNA expression levels of *ALOX5* were calculated using the 2^−ΔΔCt^ method, with GAPDH as an internal control.

### 4.6. Measurement of Glutathione (GSH)

After the TSN treatment, HepG2 cells were collected and lysed. The GSH level was determined with the commercial kit (S0053, Beyotime, Shanghai, China). The absorbance of the samples was detected at 412 nm.

### 4.7. Fluorescence Staining

The HepG2 cells were plated into confocal dishes or 6-well plates. After treating with or without TSN for 48 h, the cells were incubated in PBS containing 6 μM Liperfluo (Dojindo, Kumamoto, Japan) or 10 μM BODIPY 581/591 C11 probe (Invitrogen, Carlsbad, CA, USA) for 0.5 h. The immunofluorescent images were captured by a confocal microscope (Olympus, FV3000, Tokyo, Japan). Or, the cells were subjected to trypsinization and subsequently transferred into flow cytometry tubes for a flow cytometric analysis.

### 4.8. Animals and Treatment

The specific pathogen-free (SPF) mice were purchased from the Laboratory Animal Center of Sun Yat-sen University (Guangzhou, China) and housed in a temperature-regulated (20–25 °C) and humidity-controlled (40–70%) environment under a 12 h light/dark cycle with ad libitum access to food and water. The experiments described below were designed based on the 3Rs (replacement, refinement, and reduction principles) and followed the National Institutes of Health Guidelines on the Care and Use of Animals.

To investigate TSN-induced ferroptosis in vivo, 32 male Balb/c mice (weight, 18–20 g, 6–7 weeks) were randomly assigned into 4 groups (*n* = 8). The groups consisted of a vehicle control group (received saline containing 10% propylene glycol) and 3 groups exposed to varying doses of TSN: 5, 10, or 20 mg/kg/day. After a 7-day acclimatization, the mice were administered TSN (dissolved in saline containing 10% propylene glycol) intraperitoneally once daily for 7 days. At the end of the experiment, all the mice were deeply anesthetized via the inhalation of isoflurane (RWD Life Science, Shenzhen, China) and euthanized by cervical dislocation. Liver tissues were subsequently collected, washed with ice-cold saline, and divided. One portion was fixed in 4% paraformaldehyde for IHC and the remainder was stored at −80 °C for further analysis. Liver samples from our prior study [11] were reused in this experiment. All the experiments in this study were performed in accordance with the protocol approved by the Animal Ethical and Welfare Committee of Sun Yat-sen University (approval No.: SYSU-IACUC-2023-000086).

To investigate the role of ALOX5 in TSN-induced hepatotoxicity in vivo, 40 male C57BL/6 mice (weight, 18–20 g, 6–7 weeks) were provided from the Laboratory Animal Center of Sun Yat-sen University (Guangzhou, China). After adaptive feeding, the mice were randomly assigned into four groups (*n* = 10): an AAV–control vector–vehicle control (AAV-NC-control) group, an AAV-NC-TSN (5 mg/kg/day) group, an AAV-*Alox5*-control group, and an AAV-*Alox5*-TSN group. An AAV8 vector expressing a transcript encoding an *Alox5*-targeting siRNA and EGFP under the control of the hybrid apolipoprotein E/human alpha-1-antitrypsin (ApoE/hAAT) promoter, followed by an SV40 polyadenylation signal, was developed to downregulate *Alox5* (GeneChem, Shanghai, China). The mice were injected with AAV-NC or AAV-*Alox5* (4 × 10^11^ v.g) in 200 μL of saline via the tail vein. Two weeks later, the mice were then treated with saline with 10% propylene glycol or TSN (5 mg/kg/day) by intraperitoneal injection once daily for 7 days, and their body weights were recorded daily during this period. Prior to the terminal procedures, the mice were fasted overnight and subsequently weighed. Blood was collected from the retro-orbital plexus under isoflurane anesthesia, followed by cervical dislocation while anesthetized. The liver samples were then collected and weighed. Frozen sections were prepared and the transfection rates were quantified via fluorescence microscopy. Residual liver tissues were stored at −80 °C for further analysis. All the animal procedures complied with ethical regulations and received approval from the Animal Ethical and Welfare Committee of Sun Yat-sen University (approval No.: SYSU-IACUC-2024-001653).

### 4.9. Serum Biochemical Assay

Mouse blood was sampled, kept at room temperature for 1 h, and then centrifuged at 3000 rpm for 15 min at 4 °C to obtain serum. Hepatic function biomarkers, including aspartate aminotransferase (AST), alanine aminotransferase (ALT), lactate dehydrogenase (LDH), and alkaline phosphatase (ALP), were quantitatively analyzed using commercially available assay kits according to the manufacturers’ protocols (Guangzhou Donglin, Guangzhou, China).

### 4.10. Histopathological Examination

Liver tissues were excised and immediately fixed in a 4% paraformaldehyde solution for histological preservation (BL539A, Biosharp, Hefei, China) for 24 h. Following paraffin embedding, the slices were prepared for a histological analysis with H&E staining. A histopathological assessment of the liver tissue samples was performed by two researchers who were unaware of the details of the different groups. Liver damage was assessed with the Suzuki score criteria (Supplementary Table S4) [27].

### 4.11. IHC

Liver tissues were paraffin-embedded and cut into 3 μm sections. The liver sections were immunostained with primary and secondary antibodies. Anti-ALOX5 (1:450), TFRC (1:800), and GPX4 (1:1600) antibodies were used as the primary antibodies, which are listed in Supplementary Table S2. All the samples were observed under a Nikon ECLIPSE E100 microscope (Tokyo, Japan). ImageJ was used to quantify the percentage of positive area.

### 4.12. Malondialdehyde (MDA) Assay

An MDA assay kit (S0131, Beyotime, Shanghai, China) was used to measure the MDA level. HepG2 cells or liver tissues were homogenized in a lysis solution. After incubating the mixture in boiling water, the supernatant was collected and measured. The content was analyzed based on a standard curve and then normalized for each sample.

### 4.13. Acquisition of Related Targets

TSN-related targets were predicted with the SwissTarget database (http://www.swisstargetprediction.ch/) [28], which employs ligand-based molecular similarity approaches for target prediction. Hepatotoxic-related target genes were compiled from the Genecards (https://www.genecards.org/) and OMIM (https://omim.org/) databases [29]. Ferroptosis-related target genes were downloaded from the FerrDB database (http://www.zhounan.org/ferrdb, accessed on 21 February 2023) [30].

### 4.14. Component–Target Visualization Network Construction

The network of TSN and its associated targets was constructed using Cytoscape 3.7.1. In the network, the different colors and shapes of the nodes were used to distinguish TSN and the targets.

### 4.15. Oxidative Lipidomics Analysis

HepG2 cells were treated with 1 μM TSN for 48 h and harvested. The cell pellet was reconstituted in 100 μL of ultrapure water, followed by the addition of 200 μL of methanol/acetonitrile (1:1, *v*/*v*) with an internal standard. After being processed through freeze–thaw cycles, the supernatants were collected by centrifugation at 12,000 rpm. Eicosanoid extraction was performed using Poly-Sery MAX SPE columns. The data were collected using an ExionLC™ AD ultra-performance liquid chromatography (UPLC) system coupled to a QTRAP 6500+ tandem mass spectrometer (MS/MS) (Sciex, Framingham, MA, USA), with quantification achieved via multiple reaction monitoring (MRM). The mobile phases were acetonitrile/water (60:40, *v*/*v*) with 0.002% acetic acid (A) and acetonitrile/isopropanol (50:50, *v*/*v*) (B). The gradient elution program was set as follows: 0 min (99.9% A/0.1% B); 2 min (70% A/30% B); 4 min (50% A/50% B); 5.5 min (1% A/99% B); 7 min (1% A/99% B); and 7.1 min (99.9% A/0.1% B). Metabolite significance was determined by VIP scores derived from an orthogonal partial least-squares discriminant analysis (OPLS-DA) using MetaboAnalystR. The data were log-transformed (log2) and mean-centered before OPLS-DA. Model validation was achieved through 200 permutation tests to ensure statistical reliability.

### 4.16. Statistical Analysis

The statistical data were represented as the means ± standard deviation (SD) and analyzed using Prism (GraphPad software, San Diego, CA, USA, version 8.0.1). Statistical significance was determined using a one-way analysis of variance (ANOVA), followed by Dunnett’s multiple comparisons post hoc. A *p*-value less than 0.05 was considered to indicate statistical significance.

## 5. Conclusions

In summary, this study demonstrates that TSN induces hepatotoxicity involving ferroptosis by triggering ALOX5-driven lipid peroxidation, offering new insights into the mechanism of TSN-induced liver injury. ALOX5 emerged as a critical positive modulator of lipid peroxidation, and a knockdown of ALOX5 restored the GPX4 protein level after TSN treatment, mitigated the accumulation of lipid peroxide, and balanced ferroptosis surveillance and lipid peroxidation. These findings imply that inhibitors targeting ALOX5 could potentially attenuate the hepatotoxicity of TSN and *Fructus Meliae Toosendan*, proposing a new therapeutic strategy for HILI.

## Data Availability

The original contributions presented in this study are included in the article/Supplementary Materials. Further inquiries can be directed to the corresponding authors.

## Supplementary Materials

The following supporting information can be downloaded at: https://www.mdpi.com/article/10.3390/ph18071078/s1, Figure S1: 1H NMR spectrum of the compound toosendanin (TSN); Figure S2: 13C NMR spectra of the compound TSN; Table S1: List of the ALOX5-targeting siRNA and scrambled control siRNA sequences; Table S2: List of the primary antibodies used for Western blotting and IHC; Table S3: Real-time PCR primer sequences; Table S4: Suzuki’s histological criteria.

## Abbreviations

The following abbreviations are used in this manuscript:

5-HEPE	5-hydroxyeicosapentaenoic acid
5-HPETE	5-hydroperoxyeicosatetraenoic acid
ALOX5	5-lipoxygenase
ALT	alanine aminotransferase
AST	aspartate aminotransferase
CCK-8	Cell Counting Kit-8
DILI	Drug-induced liver injury
Era	erastin
FMT	Fructus Meliae Toosendan
Fer-1	ferrostatin-1
GPX4	glutathione peroxidase 4
GSH	glutathione
IF	immunofluorescence
LDH	lactate dehydrogenase
Lip-1	liproxstatin-1
MDA	malondialdehyde
Nec-1	necrostatin-1
TSN	toosendanin
TFRC	transferrin receptor protein 1
VIP	variable importance in projection
