# Supplementary material for: Toosendanin Induces Hepatotoxicity by Facilitating ALOX5-Mediated Lipid Peroxidation and Sensitizing Cells to Ferroptosis
Source: Pharmaceuticals (Basel). 2025 Jul 21;18(7):1078. doi: 10.3390/ph18071078 (PMC12300629; doi:10.3390/ph18071078)
Supplement: Supplementary file 1 [file pharmaceuticals-18-01078-s001.zip › pharmaceuticals-3738001-supplementary.pdf]

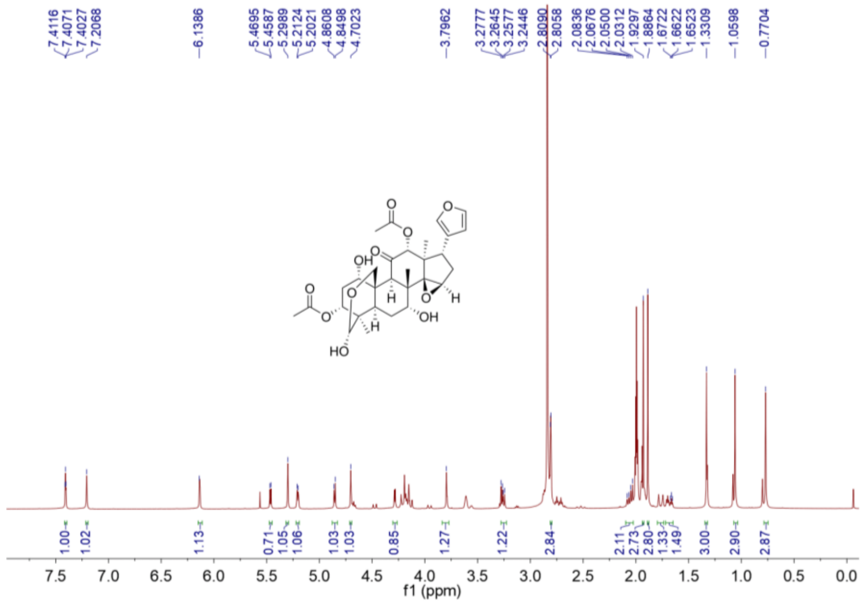

Supplemental Figure S1. ^{1}H NMR spectrum of compound toosendanin (TSN).

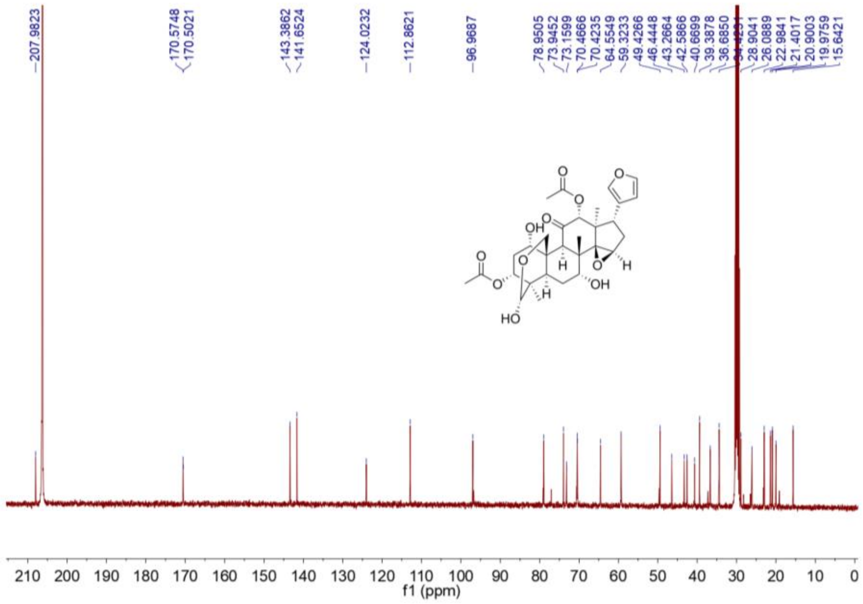

Supplemental Figure S2. 13C NMR spectra of compound TSN.

Supplemental Table S1. List of the *ALOX5*-targeting siRNA and scrambled control siRNA sequences.

Name	Forward sequence (5'-3')	Reverse sequence (5'-3')
si-*ALOX5*	GCACAGAGCUGCCCGAGAAT	UUCUCGGGCAGCUCUGUGCTT
si-NC	UUCUCCGAACGUGUCACGUTT	ACGUGACACGUUCGGAGAATT

Supplemental Table S2. List of primary antibodies used for western blotting and IHC.

Antibody	Brand	Cat. number
Rabbit anti-ALOX5	Immunoway Biotechnology (Suzhou, China)	YT0027
Rabbit anti-TFRC	Cell Signaling Technology (Danvers, MA, USA)	13113S
Rabbit anti-GPX4	Huaan Biotechnology (Hangzhou, China)	ET1706-45
Mouse anti-GAPDH	Proteintech (Wuhan, China)	60004–1-Ig
Anti-rabbit IgG, HRP-linked	Cell Signaling Technology (Danvers, MA, USA)	7074S
Anti-mouse IgG, HRP-linked	Cell Signaling Technology (Danvers, MA, USA)	7076S

Supplemental Table S3. Real-time PCR primers sequences

Gene	Forward sequence (5'-3')	Reverse sequence (5'-3')
Human ALOX5	ACAAGCCCTTCTACAACGACT	AGCTGGATCTCGCCCAGTT
Human GAPDH	TGCACCACCAACTGCTTAGC	GGCATGGACTGTGGTCATGAG

Supplemental Table S4. Suzuki's histological criteria

Grade	Congestion (%)	Vacuolization (%)	Necrosis (%)
0	None	None	None
1	Minimal (10)	Minimal (10)	Minimal (10)
2	Mild (11-30)	Mild (11-30)	Mild (11-30)
3	Moderate (31-60)	Moderate (31-60)	Moderate (31-60)
4	Severe (> 60)	Severe (> 60)	Severe (> 60)
